# Chronic Stress-Induced Neuroinflammation: Relevance of Rodent Models to Human Disease

**DOI:** 10.3390/ijms25105085

**Published:** 2024-05-07

**Authors:** Abigail G. White, Elias Elias, Andrea Orozco, Shivon A. Robinson, Melissa T. Manners

**Affiliations:** 1 Department of Biological and Biomedical Sciences, Rowan University, Glassboro, NJ 08028, USA; 2 Department of Psychology, Williams College, Williamstown, MA 01267, USA

**Keywords:** inflammation, chronic stress, UCMS, CUMS, cytokines, behavior

## Abstract

The brain is the central organ of adaptation to stress because it perceives and determines threats that induce behavioral, physiological, and molecular responses. In humans, chronic stress manifests as an enduring consistent feeling of pressure and being overwhelmed for an extended duration. This can result in a persistent proinflammatory response in the peripheral and central nervous system (CNS), resulting in cellular, physiological, and behavioral effects. Compounding stressors may increase the risk of chronic-stress-induced inflammation, which can yield serious health consequences, including mental health disorders. This review summarizes the current knowledge surrounding the neuroinflammatory response in rodent models of chronic stress—a relationship that is continually being defined. Many studies investigating the effects of chronic stress on neuroinflammation in rodent models have identified significant changes in inflammatory modulators, including nuclear factor-κB (NF-κB) and toll-like receptors (TLRs), and cytokines, including tumor necrosis factor-alpha (TNF-α), interleukin (IL)-1β, and IL-6. This suggests that these are key inflammatory factors in the chronic stress response, which may contribute to the establishment of anxiety and depression-like symptoms. The behavioral and neurological effects of modulating inflammatory factors through gene knockdown (KD) and knockout (KO), and conventional and alternative medicine approaches, are discussed.

## 1. Introduction

Chronic, unresolved stress is a major risk factor for the development of many diseases and disorders of the CNS. Many of these disorders involve an inflammatory component, which can both result from and contribute to the progression of disease [1]. This complex relationship between inflammation and chronic stress-related diseases must be further explored to better understand the etiology of disease and potential avenues for new treatment options.

Rodent models of chronic stress are commonly used to explore the molecular impact of chronic stress in the CNS. Models of chronic stress include a variety of psychologically, socially, and physically stressful events for a period of time that is considered chronic. These models result in physical and behavioral changes due to chronic stress, such as changes in avoidance behaviors; exploratory activities; grooming behaviors; or physiological changes, including weight and temperature changes [2].

The complexity and diversity of chronic stress paradigms challenge the rigor and reproducibility of these models. Behavioral models commonly differ depending on the vivarium facilities, the number of researchers, the available equipment, and the rodent strain used for these studies; however, chronic stress paradigms may be more widely variable due to the types and combinations of stressors, and the duration of the stress protocol [3]. Investigating the inflammatory effect of these chronic stress models, and their relationship to chronic stress in humans, adds a layer of complexity to selecting the best model. Therefore, it is essential to obtain direct comparative data of the different stress models and the inflammatory markers that are evaluated in each model. Many papers investigating chronic-stress-induced neuroinflammation in rodent models have measured significant changes in inflammatory mediators, including NF-κB and TLRs, and inflammatory cytokines, including TNF-α, IL-1β, and IL-6, along with others to a lesser degree. Here, we review the existing literature linking chronic stress models to inflammation known to be involved in mental health disease processes. We provide a comprehensive review of inflammatory changes found in common models of chronic stress and consider the model, duration, strain, sex, and brain regions where inflammatory changes are measured.

## 2. Chronic Stress Paradigms

There are established rodent models of chronic stress that involve physical and or psychological stressors, which result in behavioral and physiological changes [4]. This review covers the current understanding of neuroinflammation-induced chronic stress paradigms, which are known as the unpredictable chronic mild stress (UCMS) paradigm, chronic unpredictable stress (CUMS) paradigm, or chronic unpredictable stress (CUS) paradigm. These models are widely used to study the effects of chronic stress on mechanisms underlying anxiety or depression, which can be reversed through antidepressant agents [2,5]. All of these model subject mice to a variety of typically mild stressors as opposed to a single acute period of stress or prolonged repeated stress [6]. This is thought to model the accumulation of uncontrollable life stressors that are highly associated with the onset of depressive episodes [7]. The variety of stressors include physical restraint, cage tilting, water or food deprivation, cage shaking, the disruption of nesting material, forced swimming, temperature challenges, exposure to a predator odor, disruption in the light–dark cycle, static noise, social isolation, and exposure to new cage partners and can be presented in any combination or order [8,9,10,11,12,13,14,15]. The number of stressors per day can range from 1–3, while the duration of the protocol can range from 3 weeks to several months (sometimes with weekends off). Furthermore, the results can differ depending on the animal strain [16]. The high variability among protocols makes it important for investigators to include their stress schedule in publications, so comparisons between protocols can be made. Regardless of variables in chronic stress protocols, the unpredictable environmental and social micro-stressors result in behavioral and physiological changes that are evaluated using behavioral tests that measure changes in exploratory behavior or consumption in a novel environment; assess active or passive coping, learning, and memory, social interactions; or measure changes in ethological behaviors such as nesting and grooming [10,17,18]. Physiological (e.g., hypercortisolemia, hypertension, inflammatory responses, and weight loss) and behavioral (e.g., anhedonia, learned helplessness, decreased locomotor activity, and cognitive impairment) changes mirror alterations in endocrine and neural variables akin to those observed in individuals suffering from major depressive disorder (MDD) [2,19,20]. 

## 3. Chronic Stress Promotes Inflammation

Under healthy physiological conditions, certain cytokines are produced constitutively at relatively low levels for normal tissue function [21]. However, the concentration of proinflammatory cytokines significantly increases following various CNS and peripheral nervous system injuries or infections [22]. There is a bidirectional relationship between chronic stress-associated diseases and inflammation; neuroinflammation can promote diseases [23]; however, depression and anxiety can also promote neuroinflammation [24], making inflammation both the precursor to and the product of psychological diseases.

Neuroinflammation can result from inflammatory cytokines that originate from the periphery or are locally produced in the CNS. Proinflammatory cytokines generated in the periphery can enter the CNS through the blood–brain barrier (BBB). The BBB consists of endothelial cells of the capillary wall, pericytes, and astrocytes. If any of these components are compromised, circulating inflammatory cytokines, specifically IL-1β, TNF-α, and IL-6, may impede tight junction regulation in brain endothelial cells (BECs), thereby impairing BBB integrity and enhancing permeability [25]. Interactions between BECs, microglia, and astrocytes form a complex system within the brain to regulate immune cell trafficking [26]. Compromised BECs [27], microglial [28], and astrocytes [29] all have the ability to secrete proinflammatory cytokines and express receptors associated with innate immunity. Microglia constitute a first line of defense, demonstrating fast activation and recruitment to sites of damage. Secondary to microglia are astrocytes, which release inflammatory mediators that signal to microglia, ultimately perpetuating a proinflammatory response [30]. There is crosstalk between BECs and astrocytes [31]; however, research investigating the inflammatory role of BECs and the interactions between BECs and CNS cells, especially microglia, is limited. Enhancing our comprehension of these intercellular communications will lead to a better understanding of the mechanisms underlying inflammation-mediated mood disorders. The local production of cytokines in the CNS is primarily conducted by glial cells. Thus, regionalized neuroinflammation is likely due to the distribution of glial cells and region-dependent cytokine production [25]. 

**Figure 1:**
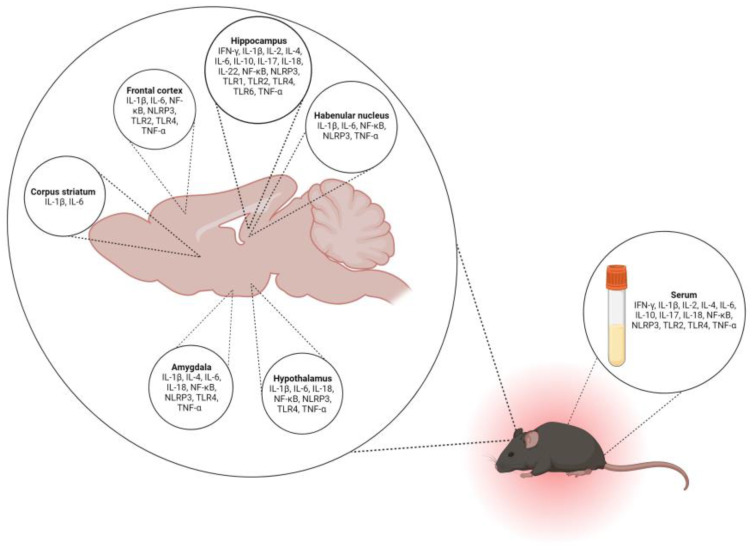
Reported inflammatory changes in brain regions and serum of rodent models of chronic stress. Following chronic stress, represented by the rodent outlined in red, the brain is dissected, serum is collected, and inflammatory factors and mediators are measured. Stress-induced inflammatory changes are reported in individual brain regions and serum. NF-κB = nuclear factor kappa-light-chain-enhancer of activated B cells, IL = interleukin, TLR = toll-like receptor, TNF-α = tumor necrosis factor-alpha, NLRP3 = NLR family pyrin domain containing 3, and IFN = interferons. Created with BioRender.com.

Rodent models of chronic stress promote inflammatory cytokines, similar to those observed in patients with MDD [32] and allow for the evaluation of brain-region-specific neuroinflammatory changes or changes in circulation (Figure 1). Below, we discuss inflammatory mediators and cytokines that are affected by chronic stress paradigms in rodents and discuss their relevance to health and disease. We focus on studies reporting the amelioration of chronic-stress-induced neuroinflammation through gene expression manipulation, and conventional or alternative medicine approaches. Alternative medicine largely exists outside of conventional health care, but some approaches are becoming increasingly integrated into the Western health-care system [33]. However, compared to conventional therapy, there is a lack of comprehensive preclinical and clinical studies on most types of alternative medicine practices. Exploring the mechanisms of both conventional and alternative medicine may reveal novel targets for antidepressant treatments. 

### 3.1. Inflammatory Mediators

In response to stress, specific proteins, including NF-κB and TLRs, manipulate the expression or activity of pro- and anti-inflammatory cytokines. In this section, we will discuss in vivo studies that investigated the effect of chronic stress paradigms on the NF-κB and TLR pathway through gene expression KD or inhibition, and conventional pharmacological or alternative medicinal approaches. Please refer to Table 1 for detailed study design information for individual chronic stress studies. 

#### 3.1.1. NF-κB

NF-κB is a transcription factor that regulates various biological processes, including cell proliferation, survival, and inflammation. It plays an important role in various autoimmune and inflammatory disorders by modulating glucocorticoid receptor signaling, corticotropin-releasing hormones, and proinflammatory cytokine release. NF-κB also plays a role in neuronal cell growth, survival, and synaptic plasticity, making it a critical factor in neurogenesis [34]. NF-κB’s prototypical proinflammatory signaling pathway can be activated through canonical or noncanonical signaling pathways. The canonical pathway is activated by several different receptors, including pattern-recognition receptors, TNF receptors, and T- and B-cells, leading to the inducible degradation of NF-κB inhibitor alpha (IκBα) and inducing the translocation of canonical NF-κB members. The noncanonical NF-κB pathway, in contrast, is activated by a distinct subset of ligands, including lymphotoxin-β receptor (LTβR), B-cell activating factor receptor, CD40 and receptor activator of NF-κB, and relies on the processing of NF-κB2 precursor protein, p100 [35]. The activation of NF-κB through either pathway induces the production of inflammatory cytokines that will be discussed in this review, making NF-κB a central mediator of proinflammatory gene induction and a focus of research studies.

Chronic stress models are associated with the hyperactivation of the NF-κB pathway in brain regions involved in stress response and affect. Elevated NF-κB signaling in the hippocampus (HPC) and lateral habenula is associated with increased depression-like behavior in rats exposed to CUS; however, this effect was reversed when rats were treated with pyrrolidine dithiocarbamate, an NF-κB inhibitor [36]. Similarly, the CUS-induced upregulation of hippocampal NF-κB is mitigated by environmental enrichment, or by fluoxetine, one of the most common selective serotonin reuptake inhibitors for MDD [37]. Weighted gene coexpression network analysis and pathway analysis revealed a gene network centered on NF-κB in UCMS *BALB/cJ* mice [38], with increased inflammatory cytokines. This highlights the importance of NF-κB as a key regulator in chronic stress and links it to antidepressant therapeutic development. 

Since NF-κB upregulation is observed in most chronic stress studies, targeting this nuclear factor via natural products used in traditional medicine has been the focus of several in vivo studies. This includes active component extracts like isofraxidin [39], curcumin [40], senegenin [41], helicid [42], trans-Cinnamaldehyde [43], ginkgo [44], and rice germ (RG) [45]; saponins [46,47] and gypenosides [48]; glycosides [49,50]; and flavonoids [51,52,53], in addition to other alkaloids, alcohols, and antioxidants like Berberine [54], geraniol [55], and ferulic acid [56]. 

Inhibition of the NF-κB pathway through KD, KO, or other regulatory mechanisms reduces risk for chronic-stress-induced behavioral changes. *NLRP3* (an inflammasome regulated by NF-κB) KO blocked NF-κB and the MAPK signaling pathway in a CUMS mouse model; this was also the case with *Neuroligin-3* KO, which inhibited the activation of NLRP3 and inactivated NF-κB in CUMS-challenged mice [12,57,58]. Overexpression of the micro-RNAs miR-204-5p or miR-182 inhibited the NF-κB pathway and alleviated depressive behavior [59,60]. NF-κB inhibition was one of the main downstream effects of TREK-1 blockage, IL-4 administration, NLRC5 deficiency, and CTRP3 overexpression, and it was associated with reduced depression-like behaviors in UCMS mice [61,62,63,64]. This makes NF-κB an important target in chronic stress and inflammation studies, particularly because this nuclear factor is upstream of many proinflammatory factors, which will be discussed in the subsections below. 

#### 3.1.2. TLRs

TLRs belong to the family of pattern-recognition receptors and play an important role in innate immunity initiation by recognizing a variety of ligands [65]. TLRs function through multiple adaptor proteins, including myeloid differentiation primary response protein 88 (MyD88) and tumor necrosis factor receptor (TNFR)-associated factor 6 (TRAF6), to activate the NF-κB pathway, leading to the increased production of proinflammatory cytokines [66]. Ten functional TLRs (TLR1-10) are expressed on peripheral immune cells, neurons, and glial cells [67]. Clinical studies have confirmed an increase in TLR expression in the brain of suicide victims and depressed patients [67], while treatment with first-line antidepressants reduced TLR expression [68]. The exact mechanisms through which chronic stress induces TLRs remains unknown; however, the unfolded protein response pathway could be involved in the activation of TLRs under stressful conditions [69]. These studies have established a strong association between increased TLR expression and chronic-stress-induced disorders. 

Chronic stress paradigms lasting from 3 to 12 weeks produce elevated levels of TLR expression, mainly in the HPC, pre-frontal cortex (PFC), blood, and serum. The TLR4-NF-κB signaling pathway seems to be the most studied TLR pathway out of the 10 TLRs and is the focus of most chronic stress in vivo studies. Wang et al. demonstrated that CUMS induces depression-like behavior and spatial memory deficits through hyperactive TLR4-NF-κB signaling and increased cytokine release, suggesting that dysregulated TLR4- NF-κB signaling may confer susceptibility to depression [70]. The siRNA-mediated KD of *TLR4* protected *ApoE*-/- mice from the negative effects of UCMS. Blocking the TLR4/NF-κB pathway significantly attenuated atherosclerotic lesions and reduced NF-κB, IκB-α, and IL-1β levels [71]. Overall, these results suggest that targeting TLR4 could be an important mechanism to reduce the inflammatory effect of UCMS and to ameliorate the maladaptive behavioral effects of stress. 

Several in vivo studies investigated the antidepressant effect of traditional medicine or alternative medicine on the TLR4 pathway. Compounds extracted from herbs and plants that effectively reversed UCMS-induced behaviors also decreased TLR4 levels in multiple brain regions. These include polysaccharides [72,73,74], polyphenols [75,76], flavonoids [77], alkaloids [78], and phytoestrogens [79,80]. Additionally, diets rich in n-3 polyunsaturated fatty acids, tryptophan, and pre- and pro-biotics show a similar antidepressant effect through TLR4 inhibition [81,82,83]. This large number of studies that confirmed the therapeutic potential of TLR4 inhibition through natural medicinal extracts suggests that this pathway plays a crucial role in inflammation and chronic stress and that targeting this pathway should be the focus of future research studies. 

When investigating the protective effect of alternative medicine on chronic stress, the results interestingly revealed the similar involvement of the TLR4 pathway in stress reduction. Aerobic exercise ameliorated the behavioral effects and restored the hippocampal function of CUMS mice. This behavioral improvement was associated with the inhibition of the stress-induced upregulation of TLR4, MyD88, and NF-κB levels, which contributed to the improvement of the hippocampal function [84]. Acupuncture prevented CUMS-induced depression-like behaviors by reversing the hyper-activation of the HMGB1/TLR4 signaling pathway and by downregulating IL-1β, TNF-α, and IL-6 in the amygdala (AMY) and blood of rats [85]. This highlights the importance of the TLR4 pathway in the context of chronic stress resilience. 

The indirect regulation of the TLR4 pathway in KD and inhibition studies has strengthened the role of TLR4 regulation in chronic stress resilience. Alirocumab, an inhibitor of PCSK9 which is a protein correlated to TLR4 regulation, prevented CUMS-induced depression-like-behaviors in *Wistar* rats. This effect of PCSK9 inhibition was associated with the inhibition of the hippocampal HMGB1/RAGE/TLR4 pathway [86]. The same antidepressant effect was observed in CUMS mice with the KD of Follistatin-like protein 1, identified as a novel inflammatory protein that alleviated depressive symptoms and microglia hyperactivation through the inhibition of the TLR4/MyD88/NF-κB pathway [87]. A few KD studies have revealed the similar involvement of additional TLRs in chronic stress resilience; KD of cyclic adenosine monophosphate response element-binding protein (*CREB*) in the HPC of UCMS mice increased resilience to stress and was associated with the downregulation of TLR1 and TLR6 [10]. Increased TLR9, and increased Clec2d, a receptor involved in sensing cellular stress, were observed in the medial prefrontal cortex (mPFC) of UCMS mice, while the KD of the latter inhibited microglial inflammation and reduced stress-induced negative emotional behaviors [88]. This indicates that other TLRs should be investigated in chronic stress and that the TLR-NF-κB pathway is one of the main mechanisms contributing to inflammation and chronic stress. There are limited studies on how CREB and other TLRs, including TLR1, 2, 5, 6, and 8, are involved in chronic stress induced neuroinflammation. Expanding our knowledge of this current research gap could lead to important pathways for developing novel treatment. 

### 3.2. Inflammatory Cytokines

Chronic stress activates the aforementioned inflammatory mediators in a manner that requires further clarification. This activation prompts microglia and astrocytes to release proinflammatory cytokines. In this section, we will discuss inflammatory cytokines that are identified most frequently in chronic stress paradigms, including IL-1β, IL-6, and TNF-α, and, to a lesser extent, IL-10, interferon-gamma (IFN-γ), IL-17, IL-22, and IL-4. Please refer to Table 1 for detailed study design information for individual chronic stress studies.

#### 3.2.1. IL-1β 

IL-1β is a proinflammatory cytokine that is activated by the NF-κB pathway. At normal levels, IL-1β expression promotes long-term potentiation and memory formation. However, at elevated levels, IL-1β becomes excitotoxic, alters synaptic activity, and modulates monoaminergic and glutamatergic synaptic transmission [89]. Elevated levels of IL-1β have been observed in MDD patients who have a poor response to first-line antidepressant treatment [90,91]. Chronic stress models in rodents demonstrate a similar pattern of inflammation, allowing for the study of antidepressant treatment.

The UCMS model produces increased levels of IL-1β after 3–12 weeks of stressors. IL-1β was directly linked to the behavioral effects of stress in an IL-1β receptor (IL-1r) KO mouse line study. *IL-1r* KO or mice overexpressing IL-1 receptor antagonist did not exhibit changes in sucrose preference or exploratory activity observed in WT UCMS mice. Additionally, *IL-1r* KO mice were protected from the inflammatory impairment in neurogenesis that occurs in UCMS. The administration of IL-1β to mice for 4 weeks significantly decreased sucrose preference, social exploration, BrdU-positive cells, and DCX-positive neurons [8]. This study provided the foundational evidence that UCMS promotes IL-1β expression and that the behavioral effects can be rescued in the absence of IL-1β. Since then, measuring IL-1β has become important in evaluating the inflammatory effect of UCMS, as has finding methods to reduce IL-1β to improve behavioral outcomes.

Currently prescribed antidepressants like fluoxetine decrease IL-1β while reversing behaviors in chronic stress models. However, combining fluoxetine treatment with a compound isolated from plants, Lilium [92], cordyceps militaris [93], or an enriched environment [37] may have a synergistic effect. Other compounds isolated from plants have similar effects, including eucommia ulmoides Oliv [49]; saikosaponin-d (SSd), a triterpene saponin [47]; isofraxidin [39]; and β-glucan, a unique agent that has immunomodulatory activity without eliciting a proinflammatory response [94,95].

Increased IL-1β levels from chronic stress can be due to changes in several upstream regulators of IL-1β, which are discussed in Section 3 of this review. NLRP3 is a pattern-recognition receptor and component of the innate immune response that mediates the expression of IL-1β in response to infection, damaged tissue, and stress. Several treatments targeting the NLRP3 pathway attenuate chronic-stress-induced IL-1β levels and improve anxiety-like and depression-like behaviors. The release of IL-1β may be dependent on the NLRP3 inflammasome-signaling pathway as *NLRP3* KO mice subjected to UCMS had reduced IL-1β in the HPC and serum [12] and were more resilient to behavioral changes. The administration of the NLRP3 inflammasome inhibitor VX-765 [96], or minocycline, a microglia inhibitor [97], before UCMS also reduced IL-1β levels. In preclinical studies, terazosin, an alpha1-adrenergic receptor antagonist and smooth muscle relaxer [98]; gelsemine [99]; and URB597, a fatty-acid amide hydrolase inhibitor [100] have also been effective in inhibiting chronic-stress-induced increases in IL-1β and reducing depression-like symptoms, similar to conventional antidepressants. These studies demonstrate that the inhibition of IL-1β decreases inflammation and protects from the behavioral effects of the UCMS model. Because of the role of IL-1β in stress and inflammation, it has become increasingly important to study the inhibition of inflammation in preclinical models. 

Another proinflammatory process to consider is inhibiting peripheral inflammatory factors from entering the brain. Dapaglifozin (Dapa), a sodium-glucose co-transporter-2 inhibitor, is thought to reinforce the BBB integrity to inhibit systemic inflammation from entering the brain and promote neuroplasticity through the NLRP3/ET-1/ETBR/BDNF/ZO-1 axis. By inhibiting peripheral inflammation into the brain, this may protect one from chronic-stress-induced inflammation. Dexamethasone (DEX) is a glucocorticoid with broad anti-inflammatory activity that also decreased IL-1β in the PFC in a UCMS model [101]. Polydatin is a glucoside derivative of resveratrol that produces anti-inflammatory effects by activating nuclear factor erythroid 2–related factor 2 (Nrf2) pathways, leading to the inhibition of NF-κB [102]. It is currently in two phase II clinical trials for traumatic/hemorrhagic shock, septic shock, and irritable bowel syndrome. There have been promising preclinical studies using polydatin in reducing UCMS-induced anxiety-like and depression-like behaviors and neuronal inflammatory changes including IL-1β levels [50]. The effect of these drugs is remarkable; however, most studies do not specifically identify central versus peripheral inflammation changes. Determining if the effect of anti-inflammatory drugs is through inhibiting the BBB penetration of systemic inflammation would shed light on these effects. 

Ultimately, IL-1β directly decreases adult hippocampal progenitor cell proliferation by arresting the cell cycle via the activation of the NF-κB pathway [103]. Ample evidence indicates that IL-1β influences all levels of the hypothalamic-pituitary-adrenal (HPA) axis, inducing the secretion of corticotropin-releasing hormone, adrenocorticotropic hormone, and glucocorticoids and reducing the sensitivity of glucocorticoid receptors. Therefore, it is possible that IL-1β is involved in the dysregulation of the HPA axis, which is one of the most robust biological markers of depression. Research suggests that increased IL-1β promotes anhedonia, learned helplessness, and memory impairment [8].

Although IL-1β expression is directly associated with chronic-stress-induced behaviors, and reduced expression reverses or prevents these behavior changes, there are currently no IL-1β targeting therapeutics. Pursuing IL-1β as a target for chronic stress-associated disorders should be considered. Of note, studies cited in this review have a wide variety of time points used for chronic stress models, ranging from 14–84 days. It seems that models as short as 21 days lead to similar effects to models that run for 84 days. Researchers conducting studies on IL-1β in the context of chronic stress may consider a shorter duration when designing their studies.

#### 3.2.2. IL-6 

IL-6 is typically regarded as a proinflammatory cytokine but has been shown to exert both pro- and anti-inflammatory properties. In the trans-signaling pathway, the soluble form of the IL-6 receptor binds to IL-6 and is transported to any cell type on which the gp130 signal-transducing chain is expressed to promote agonistic proinflammatory effects, increasing the type of cells through which IL-6 can signal. Classical IL-6 signaling produces an anti-inflammatory response through IL-6 binding to the membrane-bound cell surface receptor on subsets of T cells, neutrophils and monocytes, megakaryocytes, and hepatocytes [104]. Elevated IL-6 has been measured in patients with MDD and in the brains of rodents exposed to chronic stress models [104,105]. The release of proinflammatory IL-6 provokes neuroendocrine and neurochemical alterations that contribute to the behavioral effects of chronic stress [15]. 

Chronic stress models elevate IL-6 in multiple brain regions, blood, plasma, and serum. *IL-6* KO mice have not been evaluated in chronic stress paradigms; however, IL-6-deficient mice are resilient to the development of depression-like behaviors in the learned helplessness paradigm [106]. Therefore, IL-6-deficient rodents may also be resilient to the effects of chronic stress. 

When assessing the effect of antidepressant agents, decreased IL-6 is often associated with antidepressant effects. UCMS-exposed mice treated with gelsemine [99] or L-menthone had decreased IL-6 levels and reversed depression-like behaviors through the suppression of the NLRP3 inflammasome. The anti-inflammatory potential of quetiapine, a second-generation antipsychotic, was evaluated in a chronic stress model by Grolli et al. Quetiapine [107] and diacerein [108] significantly reduced IL-6 in the serum of chronically stressed rats, but the anti-inflammatory mechanism is largely unknown. Polydatin, which displays a strong safety and anti-inflammatory profile, rescued mice from chronic-stress-induced IL-6 and behaviors through the modulation of the NF-κB and Nrf2 pathways [50]. 

Alternative medicine approaches have also decreased IL-6 levels in models of chronic stress. These include the following reagents: prebiotic, Eucommiae cortex polysaccharides (Epss) [73]; rice germ with gamma-aminobutyric acid (GABA) [45]; theanine [45]; isofraxidin [39]; hypericum perforatum [109]; serratula coronate [109]; pumpkin extract [110]; RSNP [111]; crocin-1 [111]; bilobalide [112]; TSPG [113]; and B-glucan pretreatment [94,95]. Therapeutic approaches like acupuncture [85] and enriched environment [37] and transcutaneous vagus nerve stimulation (taVNS) [114] decreased IL-6 in either the AMY, HPC, or serum. However, behavioral tests were not completed following acupuncture or taVNS to evaluate the antidepressant-like effects. 

Though these studies strongly link decreased IL-6 levels to antidepressant behavior, completing KO or KD IL-6 studies in rodent models of chronic stress would provide stronger support for determining the role of IL-6 in the stress response. Addressing this gap would help determine if IL-6 could be targeted for the treatment or prevention of chronic-stress-associated mood disorders.

#### 3.2.3. TNF-α 

TNF-α is a proinflammatory cytokine produced in the brain by activated macrophages and T lymphocytes in response to various pathological processes. TNF-α activation occurs by binding to TNFR1 or TNFR2, which promotes the NF-κB pathway, apoptosis, and necroptosis [115]. Increased TNF-α is observed in rodent models of chronic stress and human patients diagnosed with mood disorders [15,116,117]. Rodent models of chronic stress also display functional enrichment of genes associated with immune and inflammatory responses, including the regulation of cytokine production, the TNFR1 signaling pathway, and the apoptotic TNF family pathways [118]. Excessive exposure to TNF-α can lead to a glutamate imbalance, the decreased production of neurotrophic factors, and oligodendroglia damage. This subsequently results in apoptosis and demyelination, making TNF-α one of the most important inflammatory factors to be investigated in rodent models of chronic stress [119,120,121,122]. 

One mechanism through which TNF-α can affect the CNS is by crossing the BBB. Following the stress-induced overexpression of TNF-α in the blood, plasma, and serum, TNF-α crosses the BBB via a receptor-mediated transport system, impairing the integrity of the BBB. This results in TNF-α activation in brain regions, including the HPC, PFC, and striatum [123,124]. TNF-α then stimulates corticotropin-releasing hormone production, activating the HPA axis. This interaction increases indoleamine 2,3 dioxygenase (IDO), which breaks down tryptophan, the primary precursor to 5-HT, into quinolinic acid, a potent N-methyl-D-asparate agonist and stimulator of glutamate production, impairing astrocytic function and inducing inflammation [122]. GABA signaling is one mechanism that may reduce the effects of TNF-α in the brain. GABA-enriched rice germ reduced depression-like behaviors and TNF-α in the hypothalamus (HYP) and serum of chronically stressed mice [45]. GABA cannot permeate the BBB; therefore, GABA is likely to decrease peripheral proinflammatory cytokines, eventually decreasing the levels in the brain. Following chronic stress, mice express BBB breakdown accompanied by decreased claudin-5 (Cldn5) in the HPC and increased TNF-α in the HPC and serum. Cldn5 overexpression and *EZH2* KD block the stress-induced aggregation of TNF-α in the HPC and attenuate depression-like phenotypes. Moreover, 5 mg/kg/day of fluoxetine normalized Cldn5 activation in vivo and inhibited TNF-α elevation in the blood and HPC of chronically stressed mice [125]. Together, these findings suggest that chronic-stress-induced BBB breakdown allows for the diffusion of TNF-α into the HPC, leading to the development of depression. Studies suggest that chronic stress leads to a loss of BBB integrity due to decreased P-glycoprotein [126]; however, studies also suggest that TNF-α impairs BBB integrity [127,128]. Whether TNF-α is essential for chronic-stress-induced BBB breakdown remains elusive. Therefore, the mechanism by which TNF-α infiltrates the brain may be an important process in linking systemic inflammation to diseases of the CNS.

Considering that TNF-α expression is upregulated in rodent models of chronic stress, leading to the alteration of biological processes and behaviors, studies have focused on the effects of current FDA-approved drugs on the modulation of TNF-α. The TNF-α inhibitor, infliximab [129]; the IDO inhibitor, 1-methyltryptophan; and minocycline, an inhibitor of innate immune cells, reduced TNF-α and rescued mice from the behavioral effects and neuronal damage induced by chronic stress paradigm [97]. 

Plant extracts have reduced TNF-α in animal models, including Serratula coronata, Hypericum perforatum, and Valeriana officinalis [109]; Isofraxidin [39]; SSd [47]; L-Menthone [130]; RL-118 [131]; bilobalide [112]; ginsenosides [113]; polydatin from Polygonum cuspidatum [50]; escitalopram, Dapa, and co-treatment with BQ-788 and Dapa [58]; and Lilium saponins and Fluoxetine [92]. The anti-inflammatory mechanisms of co-treatments were mediated through the inactivation of the COX-2/PGE2/IL-22 axis [92] or the promotion of NLRP3 ubiquitination via the MARCHF7 protein, ultimately inhibiting the activation of the NLRP3 inflammasome [47]. Comparable to fluoxetine, either an enriched environment or acupuncture prevented the stress-induced increase of TNF-α in the HPC, AMY, serum, and spleen of chronically stressed rats [37,85]. The same result was obtained in the serum of rats with taVNS for 2 weeks post-chronic stress [114]. 

Chronic stimulation of the innate immune response can lead to inflammation-mediated depression; however, pre-stimulation of the innate immune response has been effective in preventing depression-like phenotypes in rodent models, while 24 h pre-stress exposure to a single dose (20 mg/kg) of β-glucan prevented a chronic-stress-induced increase in TNF-α in the HPC and mPFC. Furthermore, minocycline pretreatment (40 mg/kg) abolished the β-glucan-induced decrease in TNF-α [94]. This study proposes a unique avenue of stimulating the innate immune system without initiating a proinflammatory response, to prevent the onset of depression-like behaviors. The underlying molecular mechanisms of β-glucan remain unclear, and whether this method would function in the same manner following or during the onset of depression-like phenotypes should be studied. 

In this review, we have identified that the following brain regions have CMS-induced increased TNF-α levels: the cortex, frontal cortex, PFC, HPC, CA1, dentate gyrus (DG), striatum, HYP, AMY, habenular nucleus, and mPFC. However, there may be specific brain regions that are more susceptible to increased TNF-α than others. The administration of a traditional medicine agent, Rannasangpei (RSNP), or the active ingredient crocin-1 following UCMS reduced TNF-α mRNA expression in the PFC and HPC. The reason underlying the brain-specific changes of TNF-α remains unknown but is supported by a previous study that measured the exercise-induced reduction of TNF-α in the HPC but not the PFC, though other brain regions such as the HPC and the lateral habenula are also sensitive to increased TNF-α [36,125]. Accumulating data support the sensitivity of the PFC to the detrimental effects of stress exposure [15,118,132]. However, there is limited research available investigating the role of stress-mediated TNF-α alterations in the PFC. Furthermore, RSNP and crocin-1 ameliorated stress-induced apoptosis in the HPC and PFC, and it is proposed that these treatments exert neuroprotective and anti-depressant-like effects through the BDNF pathway [111]. Due to the brain-region-specific effects of inhibiting TNF-α signaling, it may be important to identify the behavioral brain regions that are most susceptible to chronic-stress-induced TNF-α. In addition to HPC and PFC, striatum-based circuits play a critical role in the immune and inflammatory processes associated with depression. Upstream signaling events of TNF-α lead to NF-κB pathway activation in the dorsal striatum, inducing the rapid transcription of genes regulating inflammation, cell survival, proliferation, and differentiation, and subsequently depression-like behaviors [133,134]. CYLD lysine 63 deubiquitinate (CYLD) is crucial in immune response and inflammation and is highly expressed in the dorsal striatum [133,135]. *CYLD* KO mice expressed increased TNF-α in the dorsal striatum along with stress-induced anxiety-like behaviors [133]. 

To our knowledge, at the time of writing this review *TNF-α* KD studies have not been conducted in rodent models of chronic stress. Although this study has yet to be conducted at the time of writing this review, the global inhibition of TNF-α may have undesirable biological consequences. Further investigation of the pathways involving TNF-α, or brain-region-specific targeting, may better harness the therapeutic potential of this inflammatory cytokine.

#### 3.2.4. IL-10

IL-10 is an anti-inflammatory cytokine that maintains immune homeostasis by regulating the magnitude of inflammatory responses. IL-10 is produced by Th2 cells, macrophages, and CD8+ cells and inhibits the synthesis of a wide range of proinflammatory cytokines, including IFN-γ, IL-6, and TNF-α [136]. IL-10 antagonists are effective in the treatment of autoimmune diseases such as systemic lupus erythematosus, and stress has been shown to aggravate the course of this disease [109]. A correlation between distress and IL-10 levels has been suggested and further investigated using rodent models of chronic stress. However, the role of the anti-inflammatory cytokine IL-10 in chronic-stress-induced depression is not as straightforward as the proinflammatory cytokines discussed previously. Some studies have demonstrated that chronic stress decreased IL-10 in the HPC of male [37,111] and female rodents, whereas other studies have observed no effect on hippocampal IL-10 following chronic stress in male [137,138] or female [138] rodents. Furthermore, chronic stress studies have reported significantly increased [137] or decreased [138] serum IL-10 in female rodents. Xia et al. measured no difference in serum IL-10 in male rodents following chronic stress, whereas others measured a significant decrease [138,139] or increase [109]. The clarification of conflicting findings of increased, decreased, or no change in serum and HPC IL-10 levels following chronic stress could reveal possible sex differences in susceptibility to chronic-stress-induced depression. 

Treatments, including RSNP, crocin-1 [111], and an enriched environment [37], increased IL-10 in the HPC, which attenuated depression-like behaviors [9,111]. These effects are mediated through NF-κB modulation, ultimately inhibiting proinflammatory activation and promoting anti-inflammatory genes, which modulates the microglial phenotype in the HPC. Unique combinations of plant extracts decreased serum IL-10 compared to the stress group; however, the effects on depression-like behaviors need to be analyzed [109].

#### 3.2.5. IFN-γ

In humans, the production of the proinflammatory cytokine IFN-γ increases when subjects experience physiological stress. Notably, individuals who have high stress-induced IFN-γ versus negative immunoregulatory cytokines, such as IL-10, experience significantly increased symptoms of stress-induced anxiety and depression compared to subjects with higher IL-10 expression [9]. Conventional antidepressants, such as sertraline and fluoxetine, result in the upregulation of anti-inflammatory cytokine IL-10 and decrease the production of IFN-γ and thus are effective at reducing anxiety-like and depression-like symptoms in patients [121]. However, the investigation into IFN-γ in the development of depression-like symptoms is limited and has only been conducted in more recent years. 

Studies have measured increased INF-γ in the HPC [138], the CA1 [92], and the DG [59] region of the HPC. However, Xia et al. measured no change in hippocampal INF-γ expression following chronic stress in male or female rats. To note, control and stressed female rats expressed significantly less IFN-γ in the HPC compared to their respective male counterparts [137]. Findings in peripheral IFN-γ changes following chronic stress have been more consistent, with studies reporting increased IFN-γ in serum [137,138] and blood [92] compared to controls. Sh-COX-2 and lilium saponins + fluoxetine + overexpression negative control (oe-NC) prevented a chronic-stress-induced IFN-γ increase in the HPC and blood and improved negative behaviors, whereas lilium saponins + Fluoxetine + oe-COX-2 increased IFN-γ in the HPC and blood [92]. Chronic-stress-induced IFN-γ in the DG was attenuated with miR-204-5p overexpression [59]. 

#### 3.2.6. IL-17

Elevated IL-17 in the serum of patients with MDD has been reported and further investigated using rodent models of chronic stress; however, the role of IL-17 in depression remains elusive due to conflicting findings. M1 and M2 macrophage phenotypes release proinflammatory and anti-inflammatory cytokines, which induce the differentiation of Th17 and Treg. IL-17 links T-cell activation to neutrophil mobilization, mediating protective innate immunity to pathogens and contributing to the pathogenesis of inflammatory diseases, including psoriasis and rheumatoid arthritis [140]. It is believed that excessive IL-17 binds to the interleukin-17 receptor (IL-17Rc) in microglia and astrocytes further activate them, thereby inducing depression-like behaviors. Huang et al. displayed this effect by subjecting mice to chronic stress and measured increased expression of IL-17A, IL-17Rc, microglia M1 phenotype, and astrocyte A1 phenotype in the HPC. A low dose of IL-2 reduced Th17 differentiation, thereby decreasing IL-17A secretion and alleviating the expression of IL-17Rc in microglia and astrocytes, thus inhibiting their activation and attenuating depression-like behaviors [141]. Zhang et al. further supported these findings by reporting increased serum and HPC IL-17 concentration following chronic stress [138]. However Shi et al. reported significantly decreased levels of IL-17A and IL-17F in the serum of chronically stressed mice [142]. 

#### 3.2.7. IL-18

IL-18 is a proinflammatory cytokine produced in T cells, B cells, and macrophages and activated by caspase-1 cleavage in response to autoimmune, inflammatory, and infectious diseases [143]. The activation of the NLRP3 inflammasome induces pyroptosis, resulting in the leakage of proinflammatory cytokines, such as IL-18. IL-18 is upregulated in the HPC [47,49,58,144], PFC [101], serum [58], plasma [97] and HYP [45] of rodents subjected to chronic stress. Many compounds reduce IL-18 expression through the modulation of the NLRP3 pathway, resulting in attenuated depression-like behaviors. The heightened expression of IL-18 is reduced through the inhibition of the NF-κB and NLRP3 inflammasome complex via Acubin (10 or 20 mg/kg/day) [49], RG [45] DEX [101], and SSd [47]. NLRP3 function can also be modulated through the inhibition of CYP17A1 and CYP19A1 via clotrimazole (30 mg/kg/day) [144], which produces an anti-inflammatory response and reduces depression-like behaviors. The modulation of the NLRP3 inflammasome can also be achieved through the NLRP3/IL/TNF-α/miR-501-3p/ZO-1 axis via Dapa (1 mg/kg/day) [58]. Additionally, the inhibition of peripheral proinflammatory cytokines, including IL-18, and microglial inhibition via minocycline reduces depression-like behaviors [97]. Ultimately, chronic stress significantly increases IL-18 in the HPC, PFC, HYP, and periphery, which can be reduced by various treatments that suppress the expression of the NLRP3 inflammasome and attenuate depression-like behaviors. 

**Table 1:** Summary of the studies reporting inflammatory changes in the brain or blood in rodent models of chronic stress. Included in this table is the duration of chronic stress, lists of inflammatory modulators and factors that are measured in response to chronic stress paradigms, tissue analyzed, the species/sex of rodent, and the citation. This table is organized by species, and duration of stress protocol. Abbreviations: HYP = hypothalamus, HPC = hippocampus, PFC = prefrontal cortex, FC = frontal cortex, AMY = amygdala DG = dentate gyrus, NF-κB = nuclear factor kappa-light-chain-enhancer of activated B cells, IL = interleukin, TLR = toll-like receptor, TNF-α = tumor necrosis factor-alpha, MyD88 = myeloid differentiation primary response protein 88, NLRs = nucleotide-binding oligomerization domain (NOD)-Leucine Rich Repeats (LRR)-containing receptors, NLRP3 = NLR family pyrin domain containing 3, IκB-α = NF-κB inhibitor alpha, IFN = interferons, CREB = cAMP-response element binding protein, Iba-1 = ionized calcium-binding adaptor molecule 1, BDNF = brain-derived neurotrophic factor, TrkB = tropomyosin receptor kinase B, P13K = phosphoinositide 3-kinases, CamK = Ca^2+^/calmodulin-dependent protein kinase, Akt = protein kinase B, JNK = Jun N-terminal kinase, 8-iso-PGF2α = 8-iso-prostaglandin F2α, Clec2d = C-type lectin domain family 2 member D, MPK = mitogen-activated protein kinase, IDO = indoleamine 2,3-dioxygenase, IKK = IκB kinase, GPR39 = G protein-coupled receptor 39, RAGE = receptor for advanced glycation end-products, Fractalkine = CX3CL1, and SD = Sprague Dawley. The * symbol represents female rodents.

Duration (Days)	Tissue	Inflammatory Modulators	Inflammatory Factors	Species/Sex	Citation
21	FC	NF-κB	IL-1β, IL-6, and TNF-α	*C57BL/6 mice*	[70]
21	HPC	NF-κB	IL-1β, IL-6, and TNF-α	*C57BL/6 mice*	[70]
21	HPC	BDNF, p-IkBα, and Nfr2	IL-6, IL-1β, and TNF-α	*C57BL/6 mice*	[50]
24	Blood		IL-1β, IL-6, TNF-α, and IFNγ	*C57BL/6 mice*	[92]
24	CA1		IL-1β, IL-6, TNF-α, IFNγ, and IL-22	*C57BL/6 mice*	[92]
28	Serum		IL-1β	*C57BL/6 mice*	[12]
28	HPC	NLRP3, p-p65, p-JNK, and p-p38	IL-1β	*C57BL/6 mice*	[12]
28	HPC	NF-κB		*BALB/c mice* * BALB/c mice *C57BL/6 mice* * C57BL/6 mice	[38]
28	HPC	TLR4, MyD88, and NFκB	IL-1β, IL-6, and TNF-α	* BALB/c mice	[87]
28	HPC	NLRP3, caspase-1	IL-1β	*BALB/c mice*	[96]
28	Plasma		TNF-α, IL-1β, and IL-18	*BALB/c mice*	[97]
28	mPFC	Clec2d, TLR9, MyD88, NF-κB, IκBα, NLRs, and NLRP3	IL-1β	*C57BL/6 mice*	[88]
28	HPC	TLR4, NF-κB, and MyD88	IL-1β, IL-10, and TNF-α	*C57BL/6 mice*	[84]
28	HPC	NF-κB, MAPK, caspase-1, and NLRP3	IL-1β, TNF-α, and IL-18	*C57BL/6 mice*	[47]
28	HPC	IDO, BDNF	TNF-α, IL-1β, and IL-6	*C57BL/6 mice*	[15]
28	PFC	IDO, BDNF	TNF-α, IL-1β, and IL-6	*C57BL/6 mice*	[15]
28	Serum		TNF-α, IL-1β, and IL-6	*C57BL/6 mice*	[15]
28	Striatum		TNF-α, IL-6	*C57BL/6 mice*	[15]
30	HPC	BDNF	TNF-α, IL-6, and IL-10	*C57BL/6 mice*	[111]
30	PFC		TNF-α, IL-6	*C57BL/6 mice*	[111]
35	Cortex	BDNF, TLR4	TNF-α, IL-6, and IL-1β	*C57BL/6 mice*	[82]
35	HPC	BDNF, TLR4	TNF-α, IL-6, and IL-1β	*C57BL/6 mice*	[82]
35	HPC	TLR4	IL-6, IL-1β, and TNF-α	*C57BL/6 mice*	[81]
35	Serum		IL-1β	*C57BL/6 mice*	[49]
35	HPC	NLRP3, caspase-1, and p-p65	IL-1β, IL-18	*C57BL/6 mice*	[49]
35	HPC	BDNF, TrkB, and CREB	IL-1β, IL-6	*C57BL/6 mice*	[95]
35	HPC		IL-1β	*C57BL/6 mice*	[8]
35	HPC	NLRC5, NF-κB	IL-6, IL-1β, and TNF-α	*C57BL/6 mice*	[64]
35	HPC		TNF-α, IL-1β, and IL-6	*C57BL/6 mice*	[94]
35	PFC		TNF-α, IL-1β, and IL-6	*C57BL/6 mice*	[94]
35	HYP	NF-κB, NLRP3, and caspase-1	TNF-α, IL-6, IL-18, and IL-1β	*C57BL/6 mice*	[45]
35	Serum		TNF-α, IL-6	*C57BL/6 mice*	[45]
42	HPC	NF-κB, NLRP3, and IkB-α	TNF-α, IL-1β, and IL-6	*BALB/c mice*	[39]
42	HPC		TNF-α	*C57BL/6 mice*	[125]
42	HPC	NF-κB	TNF-α, IL-1β, TGF-β, and IL-10	*C57BL/6 mice*	[52]
42	HPC	GPR39, CREB, and NF-ΚB	TNFα, IL-6	*C57BL/6 mice*	[60]
42	Serum	GPR39, CREB, and NF-ΚB	TNFα, IL-6	*C57BL/6 mice*	[60]
42	PFC	HMGB1, TLR4, NF-κB, TNFR1, MyD88, IκB-α, iNOS, and TRAF2	IL-1β, TNF-α, IL-5, IL-6, IL-7, IL-9, IL-13, and IFN-γ	*C57BL/6 mice*	[79]
42	Serum		IL-1β, TNF-α	*C57BL/6 mice*	[79]
42	Serum		TNF-α	*C57BL/6 mice*	[125]
44	HPC	TLR1, TLR6		*C57BL/6 mice*	[10]
49	HPC	NF-κB, IKKα, and IκBα	TNF-α, IL-1β	*C57BL/6 mice*	[62]
56	HPC	NF-κB, IKKβ, and IKKα	IL-1β, IL-6, and TNF-α	*C57BL/6 mice*	[48]
56	HPC	TrkB, ERK, CREB, NF-κB, and NLRP3		*C57BL/6 mice*	[74]
56	PFC	TLR4, TNFR1, NK-kB, IκB-α, iNOS, and TRAF2	TNF-α, IL-1β	*C57BL/6 mice*	[80]
56	Serum		TNF-α, IL-1β	*C57BL/6 mice*	[80]
56	Serum		IL-1β, IL-6, IL-17A, and IL-17F	*C57BL/6 mice*	[142]
56	HPC	RORγt	IL-6, IL-4	*C57BL/6 mice*	[142]
63	HPC	CREB, Myd88, NF-κB, and TLR4		*C57BL/6 mice*	[83]
84	PFC	NLRP3, caspase-1	IL-1β, IL-18	*C57BL/6 mice*	[101]
23	HPC	CREB	IL-6, IL-1β	*ICR mice*	[99]
23	PFC		IL-6, IL-1β	*ICR mice*	[99]
23	HYP	BDNF, NLRP3, and CREB	IL-6, IL-1β	*ICR mice*	[99]
23	Corpus Striatum		IL-6, IL-1β	*ICR mice*	[99]
28	HPC	MCP-1, TLR4, NF-κB, and p-p38	TNF-α, IL-1β, and IL-6	*ICR mice*	[73]
28	PFC	NF-κB, NLRP3, and capsase-1	TNF-α, IL-1β, and IL-6	*ICR mice*	[56]
28	Serum		IL-1β	*ICR mice*	[56]
42	HPC	NLRP3, NF-ΚB	TNF-α, IL-1β	*ICR mice*	[41]
42	HPC	NLRP3, caspase-1	TNF-α, IL-1β, and IL-6	*ICR mice*	[130]
42	HPC	HMGB1, TLR4, and NF-κB	IL-1β, IL-6, and TNF-α	*ICR mice*	[76]
42	Serum		IL-1β, IL-6, and TNF-α	*ICR mice*	[76]
42	HPC	TLR4, p38, NF-κB, NLRP3, and caspase-1	TNF-α, IL-1β, and IL-6	*ICR mice*	[78]
42	PFC	TLR4, p38, NF-κB, NLRP3, and caspase-1	TNF-α, IL-1β, and IL-6	*ICR mice*	[78]
42	HPC		IL-6, IL-17A, IL-17Rc, and TGF-β	*ICR mice*	[141]
42	PFC	NF-κB, NLRP3	IL-1β, IL-6, and TNF-α	*ICR mice*	[55]
42	AMY	NLRP3, NF-κB	TNF-α, IL-18, IL-1β, and IL-4	*ICR mice*	[57]
42	Cortex	NLRP3, NF-κB	TNF-α, IL-18, IL-1β, and IL-4	*ICR mice*	[57]
42	HPC	NLRP3, NF-κB	TNF-α, IL-18, IL-1β, and IL-4	*ICR mice*	[57]
44	HPC	HMGB1, RAGE, IκBα, TrKb, and NF-ΚB	IL-1β, IL-6, and TNF-α	*ICR mice*	[51]
44	Serum		IL-1β, IL-6, and TNF-α	*ICR mice*	[51]
56	HPC	TLR4	IL-1β, IL-6, and TNF-α	*ICR mice*	[75]
60	HPC	NF-κB, IKKα, IKKβ, and iNOS	IL-1β, TNF-α, and IL-6	*ICR mice*	[54]
28	HPC		TNF-α, IL-1β	*SAMP8 mice	[131]
21	HPC	TLR4, NF-κB-1, p-p65, IκBα NLRP3, ASC, and caspase-1	TNF-α, IL-1β, and IL-18	*SD rats*	[43]
21	PFC	TLR4, NF-κB-1, p-p65, IκBα NLRP3, ASC, and caspase-1	TNF-α, IL-1β, and IL-18	*SD rats*	[43]
21	Serum		TNF-α, IL-1β, and IL-18	*SD rats*	[43]
21	HPC	NF-κB, FGF2		*SD rats*	[46]
28	Habenular nucleus	NLRP3, TrkB, and NF-κB	IL-1β, TNF-α, and IL-6	*SD rats*	[36]
28	HPC	NLRP3, TrkB, and NF-κB	IL-1β, TNF-α, and IL-6	*SD rats*	[36]
28	Cortex	TLR-4, NLRP3, and caspase-1	IL-1Β, TNF-α	*SD rats*	[77]
28	HPC	TLR-4, NLRP3, and caspase-1	IL-1Β, TNF-α	*SD rats*	[77]
28	Serum		IL-10	*SD rats*	[139]
28	Serum		TNF-α, IL-1β, and IL-6	*SD rats*	[85]
28	AMY	TLR4	HMGB1, IL-6, TNF-α, and IL-1β	*SD rats*	[85]
35	Serum		IL-1β	*SD rats*	[93]
35	FC	p-Akt/Akt		*SD rats*	[93]
42	HPC	NLRP3, NF-ΚB	IL-1β	*SD rats*	[53]
42	HPC	TrKB, p75, GDNF, and GFR-α1	IL-1β, IFN-γ, IL-4, IL-10, and TGF-β	*SD rats* * SD rats	[137]
42	Serum		IFN-γ, IL-1β, IL-4, IL-10, and TGF-β	*SD rats* * SD rats	[137]
49	HPC	iNOS, NF-κB	TNF-α, IL-1β, IL-6, and IL-10	*SD rats*	[37]
49	Serum		TNF-α, IL-1β, IL-6, and IL-10	*SD rats*	[37]
70	HPC	NF-κB IκB-α	IL-1α, TNF-α	*SD rats*	[61]
84	HPC	IKKβ, IκBα, iNOS, and COX2	IL-1β, TNF-α, and IL-6	*SD rats*	[42]
84	Serum		IL-1β, TNF-α, and IL-6	*SD rats*	[42]
56	HPC	TrKB	TNF-α, IL-6	*SPF mice*	[112]
56	Serum		TNF-α, IL-6	*SPF mice*	[112]
28	PFC	IDO, NF-κB	IL-1β, IL-4	*Swiss mice*	[63]
28	HPC	IDO, NF-κB	IL-1β, IL-4	*Swiss mice*	[63]
28	HPC	NLRP3, Caspase-1	IL-18	*Wistar rats*	[144]
28	Serum		TNF-α, IL-6	*Wistar rats*	[110]
35	Cortex		TNF-α	*Wistar rats*	[58]
35	HPC	NF-κB p65, NLRP3, and caspase-1	IL-1β, IL-18, and TNF-α	*Wistar rats*	[58]
35	Serum		IL-1β, IL-18	*Wistar rats*	[58]
35	DG	p65 NF-κB	TNF-α, IL-1β, and IFN-γ	*Wistar rats*	[59]
35	mPFC	NF-κB	IL-1b, IL-6, and TNF-a	*Wistar rats*	[40]
40	Serum		IL-6	*Wistar rats*	[107]
42	HPC	CX3CL1, CX3CR1, p-p38, and p-JNK	TNF-α, IL-1β, and IL-6	*Wistar rats*	[113]
42	HPC	HMGB1, RAGE, NLRP3, TLR4, and NF-κB	IL-1β, IL-2, IL-6, and TNF-α	*Wistar rats*	[86]
42	mPFC	NLRP3, pERK, p-p38α, and pAkt	IL-1β	*Wistar rats* * Wister rats	[100]
42	HPC	NLRP3, pERK, p-p38α, and pAkt	IL-1β	*Wistar rats* * Wister rats	[100]
49	HPC		IFN-γ, IL-6, IL-17, and CD11b	*Wistar rats* * Wistar rats	[138]
49	Serum		IFN-γ, IL-10, and IL-17	*Wistar rats* * Wistar rats	[138]
56	Serum		IL-6	*Wistar rats*	[108]
56	Serum		TNF-α, IL-1β, and IL-6	*Wistar rats*	[114]
56	Serum		TNF-α, IL-6, and IL-10	*Wistar rats*	[109]
91	Blood		IL-1β, IL-37, and IL-38	*Wistar rats*	[44]
91	Cortex		8-iso-PGF2α	*Wistar rats*	[44]
21	Serum	TLR4, MyD88, NF-κB, and TrkB	IL-6, TNF-α	*Unknown*	[72]
21	HPC	TLR4, MyD88, NF-κB, and TrkB	IL-6, TNF-α	*Unknown*	[72]
84	Blood	TLR4, NF-κB, and IκB-α	IL-1β, TNF-α	*Unknown*	[71]

#### 3.2.8. IL-22

IL-22 is a pivotal inflammatory factor regulated by PGE2 in immune cells, including Th cells and innate lymphocytes, and can activate glial cells, leading to the production of proinflammatory cytokines [145,146]. Moreover, IL-22 has been linked to several conditions involving inflammatory tissue pathology such as Alzheimer’s disease and multiple sclerosis [146], and, more recently, depression. IL-22 can activate glial cells. Ma et al. measured depression-like behaviors and increased IL-22 in the CA1 region of the HPC of chronically stressed mice and mice with IL-22-oe. These effects were reversed along with reduced microglial activation, neuroinflammation, and neuronal damage, through Longya Lilium combined with fluoxetine treatment, which inhibits the COX-2/PGE2/IL-22 axis [92]. Limited studies explore the role of IL-22 in depression through COX-2 inhibition, which may serve as a potential antidepressant treatment. 

#### 3.2.9. IL-4

IL-4 is a multifunctional pleiotropic cytokine produced mainly by activated T cells but also by mast cells, eosinophils, and basophils. IL-4 regulates cell proliferation, apoptosis, and the expression of genes in various cell types, including lymphocytes, macrophages, fibroblasts, epithelial, and endothelial cells. It is generally accepted that IL-4 exerts anti-inflammatory effects through the suppression of the proinflammatory milieu; however, there are inconsistent reports on changes in IL-4 in depression, and the data are limited compared to other cytokines. Thus far, studies report chronic-stress-induced increased IL-4 in the HPC [63,142] and serum [137] and decreased IL-4 in the PFC [63]. The single intranasal administration of recombinant IL-4 (1 ng/mouse) decreased endogenous IL-4 in the HPC, increased IL-4 in the PFC, and ameliorated depression-like behaviors through the modulation of neuroinflammation and oxidative stress [63]. It appears that IL-4 can exert both pro- and anti-inflammatory effects depending on the region of expression. The modulation of IL-4 in various brain regions and its impact on depression-like behaviors should be further explored. 

## 4. Conclusions

It is clear that chronic stress promotes inflammation, and this can lead to the progression of disease. Utilizing chronic stress paradigms has helped identify inflammatory mediators and inflammatory cytokines, which appear to be important for the behavioral and neurological effects of chronic stress. Though more research is needed to fully understand the relationship between inflammation and chronic stress, inflammation appears to be a central player in establishing and promoting the progression of diseases, and chronic stress models produce inflammatory responses reminiscent of individuals with depression, anxiety, and similar affective disorders.

Although inflammation occurs in a variety of brain regions in response to chronic stress, the data presented here provide us with insight into brain regions that have been the focus of chronic stress studies. The brain regions that may be the most susceptible to chronic-stress-induced inflammation are the HPC and, to a lesser extent, the PFC and frontal cortex. Stress-induced inflammation was also found in the habenular nucleus, AMY, and HYP; however, there is a gap in knowledge of these areas as limited data support the involvement of these brain regions in stress-induced inflammation. Given the known role of the hyperactivity of the HPA axis in chronic stress and the production of hypothalamic cytokines TNF-α, IL-1β, and IL-6 in somatic diseases (e.g., cancer cachexia, hypertension, obesity, type II diabetes) [147], the role of the HYP in stress-induced inflammation should be further investigated. Additionally, the AMY is largely associated with psychiatric disorders, including depression and anxiety; however, the relationship between chronic stress, inflammation, and the establishment of depression-like behaviors is not well studied. Likewise, the role of the habenular nucleus in behavioral functions and emotional processes is supported, but the effects of stress-induced inflammation in this brain region remain elusive. Overall, determining the inflammatory effect of chronic stress in understudied brain regions important in mood and behavior is an important step to understanding the overall impact of stress.

We provide a comprehensive table that can be used by researchers as an experimental design tool (Table 1). Chronic stress paradigms can be highly variable, with different stressors, modifications, and mouse strains used between laboratories. Despite these variables, protocols between laboratories produce reproducible changes in inflammatory factors. Chronic stress paradigms consistently increase TNF-α, IL-1β, IL-6, NF-κB, and TLR4. Here, we highlight that one common variable between protocols is the duration of stressors, which is an important consideration for investigators. For example, TNF-α, IL-1β, and IL-6 are all upregulated in 3 week-long or longer paradigms. Researchers focused on targeting these factors could consider this time factor in their study design. Overall, it is encouraging that changes that occur in the human brain can be recapitulated through a variety of chronic stress protocols in rodents.

Our current understanding of the mechanisms by which chronic stress induces inflammation remains very limited, and more research is required to elucidate the complex relationship between stress and inflammation, as well as the specific effects on depression-like behaviors. Additional limitations to research in this field arise from variables in the reporting and measurement of inflammatory factors; measuring mRNA versus protein, brain regions or serum levels, and the timing of the measurement post chronic stress should be considered. Here, we present studies that include these experimental details in their reports, in an effort to overcome barriers to understanding the mechanisms of stress-induced inflammation. 

We have summarized a wide-range, but by no means exhaustive, number of studies documenting the role of chronic stress inflammatory mediators and factors on the establishment of depression-like behaviors. The studies described above collectively illustrate a crucial role for NF-κB, TLRs, TNF-α, IL-1β, IL-6, and other cytokines in the regulation of chronic-stress-induced periphery and CNS inflammation, with measurable downstream effects on behavior and neurogenesis. There are inflammatory mediators that are less commonly reported in the literature. The downregulation of the inflammatory mediator CREB has been shown to promote neurogenesis, prevent behavioral effects, and decrease the expression of genes associated with the immune system and inflammation [10], but these effects and the role of CREB on proinflammatory factors in models of chronic stress are not frequently reported. TLR4 is the most studied TLR concerning chronic-stress-induced inflammation, with limited data reporting the role of TLR1, 2, 5, and 6. It is currently unclear if there are inconsistencies with the upregulation of these inflammatory factors or if researchers less commonly measure changes in these factors. Regardless, there may be additional inflammatory factors that are important to study in chronic stress paradigms that are currently under evaluated.
